# Special Issue—Biosensors and Neuroscience: Is Biosensors Engineering Ready to Embrace Design Principles from Neuroscience?

**DOI:** 10.3390/bios14020068

**Published:** 2024-01-29

**Authors:** Grace M. Hwang, Aleksandr L. Simonian

**Affiliations:** 1 Johns Hopkins University Applied Physics Laboratory, 111000 Johns Hopkins Road, Laurel, MD 20723, USA; 2 National Science Foundation, 2415 Eisenhower Avenue, Alexandria, VA 22314, USA; asimonia@nsf.gov

## 1. Introduction

In partnership with the Air Force Office of Scientific Research (AFOSR), the National Science Foundation’s (NSF) Emerging Frontiers and Multidisciplinary Activities (EFMA) office of the Directorate for Engineering (ENG) launched an Emerging Frontiers in Research and Innovation (EFRI) topic for the fiscal years FY22 and FY23 entitled “Brain-inspired Dynamics for Engineering Energy-Efficient Circuits and Artificial Intelligence” (BRAID) [1]. Since its beginning in 2007, the EFRI program has become the flagship biennial topic competition hosted by NSF/ENG/EFMA [2], receiving hundreds of topic suggestions from the global scientific and engineering communities. Each EFRI cycle typically results in two new topics determined through a rigorous external Blue Ribbon Panel process [3]. EFRI topics are intended to attract investment in transformative interdisciplinary or convergence research that could lead to new areas of fundamental or applied research and new industries or capabilities, with the aim of strengthening the United States’ leadership by making significant progress in addressing national needs and grand challenges.

The establishment of the FY22/23 EFRI BRAID was motivated by the realization that understanding the dynamics and architecture of the brain could lead to extremely energy-efficient engineering solutions, considering that the brain, comprising 100 trillion synapses, consumes around 12–20 W [4,5,6,7], which is up to six orders of magnitude more efficient than its von Neumann counterpart [8,9,10]. More explicitly, 100 trillion transistors operating on a von Neumann complementary metal–oxide–semiconductor (CMOS) device would consume well over 1 MW [11]. In addition to power and data efficiency, the embodied brain is known to learn flexibly in time-varying contexts [12] and across many spatial and temporal scales [13], in part through different types of sparsity [14,15,16]. Impressively, animals can learn from a single exposure [17], learn continually or causally throughout their entire lifespan, and generalize from few examples across different contexts; these properties of biological learning collectively surpass the capacities of modern AI techniques [12,16,18,19,20].

Because EFRI BRAID was launched in 2021, it could leverage knowledge and data generated by major prior or ongoing funding activities from the United States, including the Brain Research Through Advancing Innovative Neurotechnologies^®^ (BRAIN) Initiative [21,22], the Intelligence Advanced Research Projects Activity’s (IARPA) Machine Intelligence from Cortical Networks (MICrONs) project [23], and the EU’s Human Brain Project [24].

In addition to addressing two distinct grand challenges [25,26], BRAID sought to advance the application of a new class of brain-inspired engineering learning system based on novel or existing neuroscience theories [27,28,29,30,31] in the domain of autonomous systems, including neuromorphic sensors [32,33,34], brain-inspired robots [35,36,37], metacontrollers for multi-agent robots [38,39,40], neuromorphic medical technologies [41,42], and other applications with global economic and health benefits. BRAID especially focused on improving performance metrics beyond energy- and data-efficient learning algorithms [20,43] and learning hardware, including the development of neuromorphic systems [10,44,45,46], the designs of which were based on insights from recent neuroscience advances [27,31,47,48,49,50,51,52]. A detailed list of BRAID awardees can be found on the public NSF search website (https://www.nsf.gov/awardsearch/advancedSearch.jsp, accessed on 1 January 2024) by entering “EFRI BRAID” as a keyword in the Advance Search tab; these search results are summarized in Table 1.

NSF’s Biosensing Program, initiated under the FY 2009 EFRI topic entitled “BioSensing and BioActuation: Interface of Living and Engineering Systems (BSBA)” [53], is committed to conducting groundbreaking research that tackles fundamental and substantial challenges, with a vision of long-lasting influence. This endeavor often involves the innovative integration of bio-inspired engineering and sophisticated technologies to meet national engineering and technological needs. In neuroscience, biosensors have become crucial, offering unparalleled insights into the intricate workings of the brain. Their value stems from their ability to provide real-time, precise measurements of various neural activities, such as neurotransmitter levels and electrical signals, in both controlled and real-world settings. This development has revolutionized our understanding of neural processes and greatly enhanced our knowledge of brain functionality and disorders. Biosensors have been key to developing novel treatments for neurological disorders, playing a pivotal role in both academic research and clinical applications. By converging with other technologies like imaging and computational analysis, biosensors have moved to forefront of research for developing comprehensive approaches to studying and treating neurological diseases, reinforcing their critical importance in the field of neuroscience.

This Special Issue on Biosensors and Neuroscience was inspired by BRAID in the context of the NSF’s Biosensing Program. Our understanding of how the brain functions has improved due to advances in modern invasive and non-invasive brain imaging, stimulation, and neuromodulation technologies. Similarly, advances in the monitoring, identification, and quantification of relevant biomarkers have been accelerated by innovations at the intersection of engineering, neuroscience, and information technology.

We invited authors to submit articles that describe advances in biosensor technology to accelerate the development of knowledge regarding brain function, as well as advances in our understanding of how the brain computes and learns to accelerate biosensor design. At the outset, we had hoped to receive submissions that focused on this reciprocal interaction between neuroscience and biosensor design, but there were no submissions that applied insights from the brain to the design or creation of next-generation biosensors, although several articles took interesting interdisciplinary approaches. One submission developed a non-invasive optical sensor that, when combined with machine learning algorithms, could detect blunt force events in the brain (contribution 1), while other submissions focused on using biosensors as diagnostic or characterization tools for the detection of early-stage disease (contributions 2 and 3) or to provide real-time closed-loop in vivo feedback (contribution 4) in neural systems. Two submissions focused on improving electrode design from in vitro to in vivo settings for chronic use (contributions 5 and 7), while another investigated the challenges around using coatings in in vivo studies of the gut–brain axis (contribution 6). One submission evaluated an invasive approach to multiplexed protein detection across multiple tissue types (contribution 8), and another provided a tutorial on electrochemical-based biosensors (contribution 9).

## 2. Overview of Published Articles

The study by Zhuang et al. (contribution 1) describes the development of a non-invasive smart helmet to predict mild traumatic brain injury (mTBI) events in real time by embedding a femtosecond laser-fabricated fiber Bragg grating (FBG) sensor into a groove on the outer surface of a football helmet. This design allows for continuous monitoring of the wavelength of reflected light to determine the Bragg wavelength shift, which corresponds to changes in the strain applied to the FBG sensor. Such changes in strain can cause the Bragg wavelength to be expanded or compressed. Preliminary tests revealed distinctive “fingerprint” patterns from the measured transient oscillatory signals based on the impact magnitudes, directions, and latitudes. When signals from the FBG sensor are combined with machine learning techniques, the helmet can monitor complex signals generated by blunt force impact in real time during game play (or military service) in two operating modes: wired and wireless. Indeed, the research team was able to compute the physical deformation parameters of the helmet, and thus, infer the impact details, which they then used to train a suite of machine learning models for predicting blunt force events. mTBI events are difficult to measure in real time and often go undetected. Improved clinical outcomes are achieved if treatment is administered within 60 min of the injury—this period is often referred to as the “golden hour”. Thus, contribution 1 represents an excellent example of how novel unobtrusive sensors can be used to monitor individuals in their normal environment while potentially improving clinical care.

The study by Hong et al. (contribution 2) focuses on the development of a non-invasive electrophysiological recording (ERG) technique to detect early-stage retinal ganglion cell (RGC) deterioration in mice. The researchers employed a three-electrode ERG system with a programmable optogenetics stimulation device. For each mouse eye, two coil-shaped silver wires (one as the working electrode and one as the reference electrode) were placed on the corneal surface, while an LED was placed at a fixed distance in front of the test eye. The system was validated and tested using wild-type mice expressing red-light-sensitive opsins induced through adeno-associated virus injection. The researchers collected the ERG signals and developed algorithms to extract specific characteristics from the ERG waveform (a-wave, b-wave, oscillatory potentials on the rising face of the b-wave, and a slow negative response) under different test conditions. Rather than using traditional analysis methods from mixed ERG signals, they identified high-frequency components in ERG signals, specifically oscillatory potentials, as sensitive markers for detecting RGC activities. This discovery could serve as a promising non-invasive diagnosis marker for early-stage RGC degeneration in glaucoma.

The study by Monge et al. (contribution 3) discusses a novel ex vivo technique to identify protein aggregates, including tau-paired helical filaments and amyloid-β (Aβ) plaques, as biomarkers of neurodegeneration for disease diagnosis and treatment. The researchers previously tested synthetic polyelectrolytes known as oligo-p-phenylene ethynylenes (OPEs), and found that small OPEs can selectively detect fibrillar and pre-fibrillar aggregates of amyloid proteins in vitro. In this study, they systematically characterized two OPEs (anionic OPE_1_^2−^ and cationic OPE_2_^4+^) to determine their suitability as ex vivo amyloid sensors. Brain tissues from wild-type and transgenic mouse and rat models of Alzheimer’s disease and post-mortem brain tissues from humans with frontotemporal dementia–tauopathy were used. While the anionic OPE_1_^2−^ outperformed the cationic OPE_2_^4+^ in the mouse model and human tissues, it underperformed in the rat model. The research team also evaluated both OPEs’ cell toxicity and did not observe any detrimental impact at 10 μM. Most importantly, they observed that 10 μM OPEs outperformed Thioflavin T (ThT), a gold-standard dye; ThT typically operates best at 1.56 mM, which suggests that OPEs confer an improvement in sensitivity of two orders of magnitude compared to ThT. Because OPEs readily bind protein amyloids that are not toxic to cells in vitro and ex vivo at low concentrations, they may represent a promising tool for achieving the selective and rapid identification of biomarkers in vivo for the detection of general amyloidogenic disorders.

The study by Chernove et al. (contribution 4) recognizes the need for devising a closed-loop system for deep brain stimulation (DBS) for patients with Parkinson’s disease (PD). Although DBS is a well-known technique for treating PD patients, the current state of practice employs an open-loop system in which the optimal stimulation waveform is preprogrammed into the stimulator without real-time feedback (i.e., open loop). The research group hypothesized that DBS-induced glutamate release could serve as real-time feedback to enable the creation of a closed-loop DBS system. They explored whether DBS of the subthalamic nucleus (STN) would increase the rate of glutamate release in the STN and globus pallidus (GP), and whether 6-hydroxydopamine (6-OHDA)-induced nigrostriatal lesions alter the patterns of glutamate levels during DBS in the STN and GP, using a hemi-parkinsonian rat model. The authors found that the glutamate response to DBS in the STN was altered in the GP in the 6-OHDA-treated rats, which suggests that the GP site could be monitored to achieve real-time feedback for a closed-loop DBS system. However, they also raised the concern that human GP is partitioned into GPe and GPi, with GPi being a common clinical target. In contrast, GP in rat is more homologous to GPe. This contribution is an excellent example of how animal models and real-time in vivo biosensors can guide the design of next-generation neuromodulation technologies to improve clinical outcomes for humans.

The study by Gupta et al. (contribution 5) characterizes fast-scan cyclic voltammetry (FSCV) electrode behavior on an all-diamond boron-doped diamond microelectrode (BDDME) and traditional carbon fiber microelectrodes (CFME), with the aim of optimizing the detection of these technologies for future in vivo use to monitor the concentrations of common neurotransmitters. Biofouling, a process by which proteins or other biological moieties adsorb onto the surface of electrodes, is known to be a major problem for CFME. Thus, this study additionally characterizes the in vitro biofouling properties of both BDDME and CFME. The research team used serotonin as the exemplar analyte and found that BDDME exhibited lower electrode fouling compared to CFME under the tested conditions (increasing or changing the switching potentials, frequency, analyte concentrations). CFMEs, however, showed superior sensitivity for serotonin while exhibiting a linear response at lower concentrations in all the tested conditions. The study by Hadad et al. (contribution 7) is similar to contribution 5 in that the authors also developed an in vitro FSCV assay to measure neurotransmitters using CFME. Unlike contribution 5, this group chose to characterize cortisol. Collectively, these contributions are great examples of guiding future modifications of electrode fabrication geometries or waveform selection strategies to optimize their performance for in vivo chronic biosensing applications.

The study by Girardi et al. (contribution 6) delves into the gut–brain axis, examining how gut microorganisms interact with the central nervous system (CNS) via vagal afferent neurons (VANs). Addressing the challenges of in vivo studies, the researchers developed a VAN culture model to analyze the influence of gut effector molecules on neuronal behavior. Their research, focusing on the effects of surface coatings and culture media on VAN regeneration, highlighted that Matrigel coating significantly boosts neurite growth. Additionally, their use of calcium imaging and electrophysiological recordings revealed diverse responses of VANs to effector molecules like cholecystokinin, serotonin, and capsaicin. This contribution significantly advances the development of new methods for assessing the impact of effector molecules on VAN activity, thus advancing our understanding of gut–brain interactions.

The study by Meah et al. (contribution 8) introduces an invasive approach called multiplex in situ tagging (MIST) to quantifying protein expression at single-cell spatial resolutions. This approach can detect 100 protein markers with high sensitivity across multiple tissue types without suffering from bias sampling (e.g., whereby signals are dominated by proteins in high abundance) commonly encountered in techniques like mass spectrometry. Taking cells from brain and kidney sections, the research group demonstrated MIST at single-cell spatial resolutions while co-localizing cells and protein detection in the same array. They found that eight-arm polyethylene glycol (PEG) at a 30% concentration substantially improved the spatial resolution without extending the time to detection. This contribution substantially advances biological investigation across diverse tissue types.

The review by Smutok and Katz (contribution 9) explains the difference between electrochemical-based biosensors and traditional analytical methods while highlighting different methods of reading out a sandwich-type immunoassay. Different receptor immobilization approaches, theoretical considerations of immobilized biomolecular systems, and signal-transducing mechanisms are also discussed.

## 3. Conclusions

This compilation of articles on the intersection of biosensors and neuroscience confirms the importance of biosensors in creating next-generation closed-loop adaptive systems for early-stage disease diagnosis and treatment. However, the majority of the design principles do not incorporate insights from neuroscience, which signals that a gap remains in biologically-inspired design and neuromorphic engineering research. The fields of neurotechnologies, biosensing, and neuromorphic engineering have historically operated in parallel with few points of intersection [42]. It is time to encourage the convergence of these fields to create next-generation devices with extreme energy/data efficiency, improved limits of detection, and resilience/adaptiveness. We hope the community will take advantage of opportunities to develop new biologically-inspired design principles, including an NSF Dear Colleague letter jointly issued by three directorates calling for bioinspired design collaborations to accelerate the discovery–translation process [54], among other government programs offered by the NSF Convergence Accelerator [55]. Some readers may also be interested in the FY24/25 NSF EFRI topic entitled “Biocomputing through EnGineering Organoid Intelligence (BEGIN OI)” [56], which was released in fall of 2023. The convergence of biosensors, microphysiological systems or engineered organoid systems, biology, neuroscience, neurotechnologies, neuromorphic engineering, optics, and microelectronics remains an exciting and promising area of research for advancing safe and secure healthcare technologies including brain health, and flexible and efficient artificial intelligent systems.

## Figures and Tables

**Table 1:** NSF ENG EFRI BRAID awardees.

Award #	Abbreviated Title	Principal Investigator
2223495	Optical Neural Co-Processors for Predictive and Adaptive Brain Restoration and Augmentation	Arka Majumdar ^1^
2223725	Using Proto-Object Based Saliency Inspired By Cortical Local Circuits to Limit the Hypothesis Space for Deep Learning Models	Ralph Etienne-Cummings ^1^
2223793	Unsupervised Continual Learning with Hierarchical Timescales and Plasticity Mechanisms	Gianfranco Doretto ^1^
2223811	Rapid contextual learning in resilient autonomous systems	Thomas Cleland ^1^
2223822	Neurally Inspired, Resilient Closed Loop Feedback Control of Learned Motor Dynamics	Vikash Gilja ^1^
2223827	DenPro3D—Dendritic Processing of Spike Sequences in Biological and Artificial Brains	Kwabena Boahen ^1^
2223839	Principles of sleep-dependent memory consolidation for adaptive and continual learning in artificial intelligence	Maksim Bazhenov ^1^
2317706	Efficient Learning of Spatiotemporal Regularities in Humans and Machines through Temporal Scaffolding	Dhireesha Kudithipudi ^2^
2317974	Emulating Cerebellar Temporally Coherent Signaling for Ultraefficient Emergent Prediction	Mark Hersam ^2^
2318065	Brain-inspired Algorithms for Autonomous Robots (BAAR)	Junmin Wang ^2^
2318081	Resilient autonomous navigation inspired by the insect central complex and sensorimotor control motifs	Floris van Breugel ^2^
2318101	Neuroscience Inspired Visual Analytics	Vijaykrishnan Narayanan ^2^
2318139	Fractional-order neuronal dynamics for next generation memcapacitive computing networks	Fidel Santamaria ^2^
2318152	Scalable-Learning Neuromorphics	Dmitri Strukov ^2^

^1^ Fiscal Year 2022. ^2^ Fiscal Year 2023.
